# Direct and indirect targets of carboxyatractyloside, including overlooked toxicity toward nucleoside diphosphate kinase (NDPK) and mitochondrial H^+^ leak

**DOI:** 10.1080/13880209.2023.2168704

**Published:** 2023-02-17

**Authors:** Andrzej M. Woyda-Ploszczyca

**Affiliations:** Department of Bioenergetics, Faculty of Biology, Adam Mickiewicz University, Poznan, Poland

**Keywords:** ADP/ATP carrier (AAC), atractyloside, cattle, cockleburs, hepatotoxicity, mitochondria, nephrotoxicity, oxidative phosphorylation, Traditional Chinese Medicine, uncoupling protein (UCP)

## Abstract

**Context:**

The toxicity of atractyloside/carboxyatractyloside is generally well recognized and commonly ascribed to the inhibition of mitochondrial ADP/ATP carriers, which are pivotal for oxidative phosphorylation. However, these glycosides may 'paralyze' additional target proteins.

**Objective:**

This review presents many facts about atractyloside/carboxyatractyloside and their plant producers, such as *Xanthium* spp. (Asteraceae), named cockleburs.

**Methods:**

Published studies and other information were obtained from databases, such as 'CABI - Invasive Species Compendium', 'PubMed', and 'The World Checklist of Vascular Plants', from 1957 to December 2022. The following major keywords were used: 'carboxyatractyloside', 'cockleburs', 'hepatotoxicity', 'mitochondria', 'nephrotoxicity', and '*Xanthium*'.

**Results:**

In the third decade of the twenty first century, public awareness of the severe toxicity of cockleburs is still limited. Such toxicity is often only perceived by specialists in Europe and other continents. Interestingly, cocklebur is among the most widely distributed invasive plants worldwide, and the recognition of new European stands of *Xanthium* spp. is provided here. The findings arising from field and laboratory research conducted by the author revealed that (i) some livestock populations may instinctively avoid eating cocklebur while grazing, (ii) carboxyatractyloside inhibits ADP/GDP metabolism, and (iii) the direct/indirect target proteins of carboxyatractyloside are ambiguous.

**Conclusions:**

Many aspects of the *Xanthium* genus still require substantial investigation/revision in the future, such as the unification of the Latin nomenclature of currently distinguished species, bur morphology status, true fruit (achene) description and biogeography of cockleburs, and a detailed description of the physiological roles of atractyloside/carboxyatractyloside and the toxicity of these glycosides, mainly toward mammals. Therefore, a more careful interpretation of atractyloside/carboxyatractyloside data, including laboratory tests using *Xanthium*-derived extracts and purified toxins, is needed.

## Introduction

**Figure 1.**
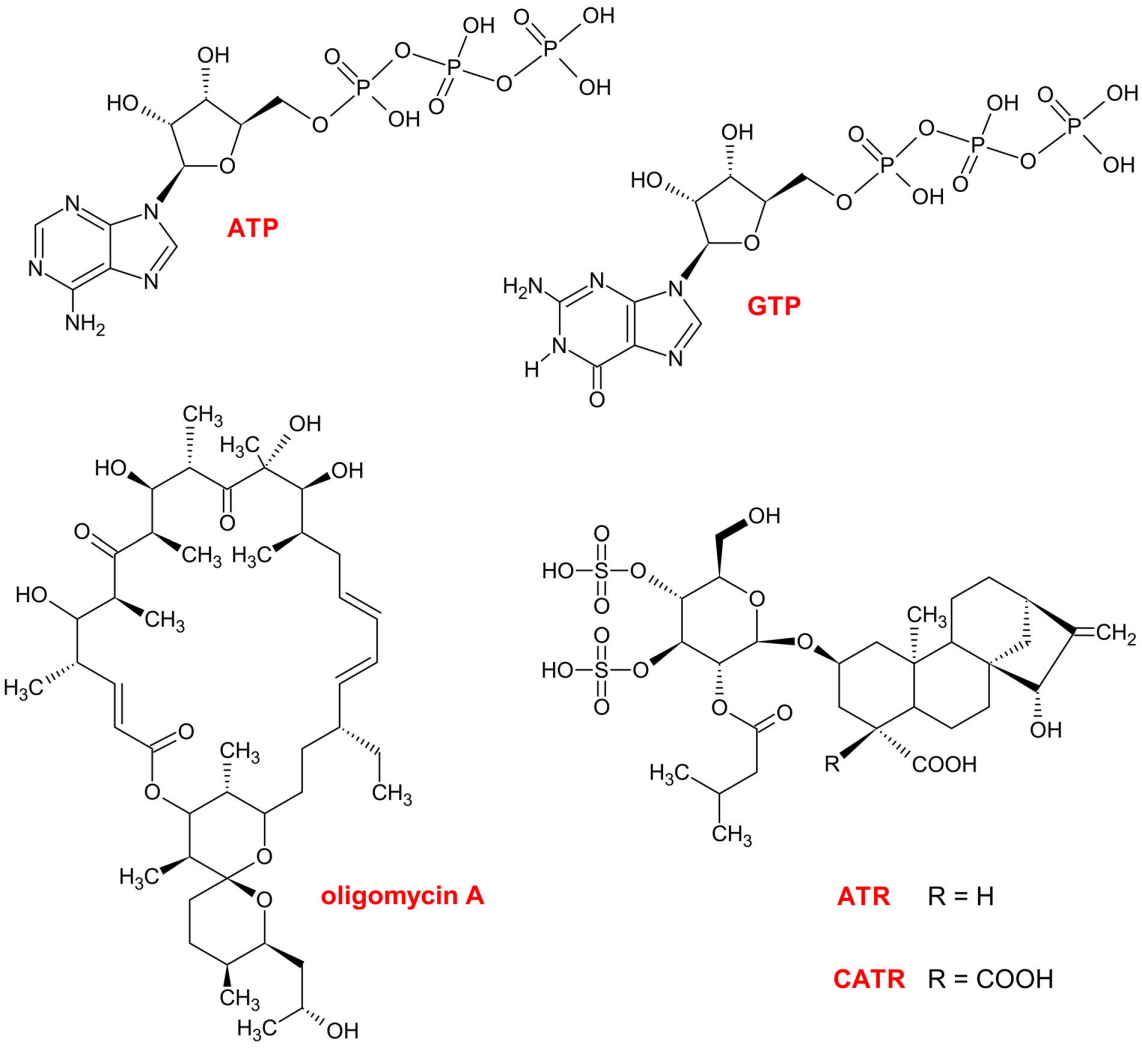
Chemical formulas. **ATP**: adenosine triphosphate, a transportable substrate of ADP/ATP carrier (AAC) and a phosphate donor, e.g., for mitochondrial nucleoside diphosphate kinase (mtNDPK). **GTP**: guanosine triphosphate, a 'diagnostic' inhibitor of uncoupling protein (UCP); guanosine diphosphate (GDP) possesses two phosphate groups and is a rather 'ambiguous' physiological blocker of UCP but can be an acceptor of a single phosphate from the mtNDPK phosphoenzyme intermediate. **ATR**: atractyloside, the stereo-isomer epi-ATR has an equatorial carboxyl group similar to **CATR**, carboxyatractyloside, with two COOH groups located at C-4′ of the *ent*-kaurane framework, and these molecules are classic inhibitors of AAC. **Oligomycin A**: an inhibitor of F_O_F_1_-ATP synthase. The figure was created by the author with ChemSketch.

**Figure 2.**
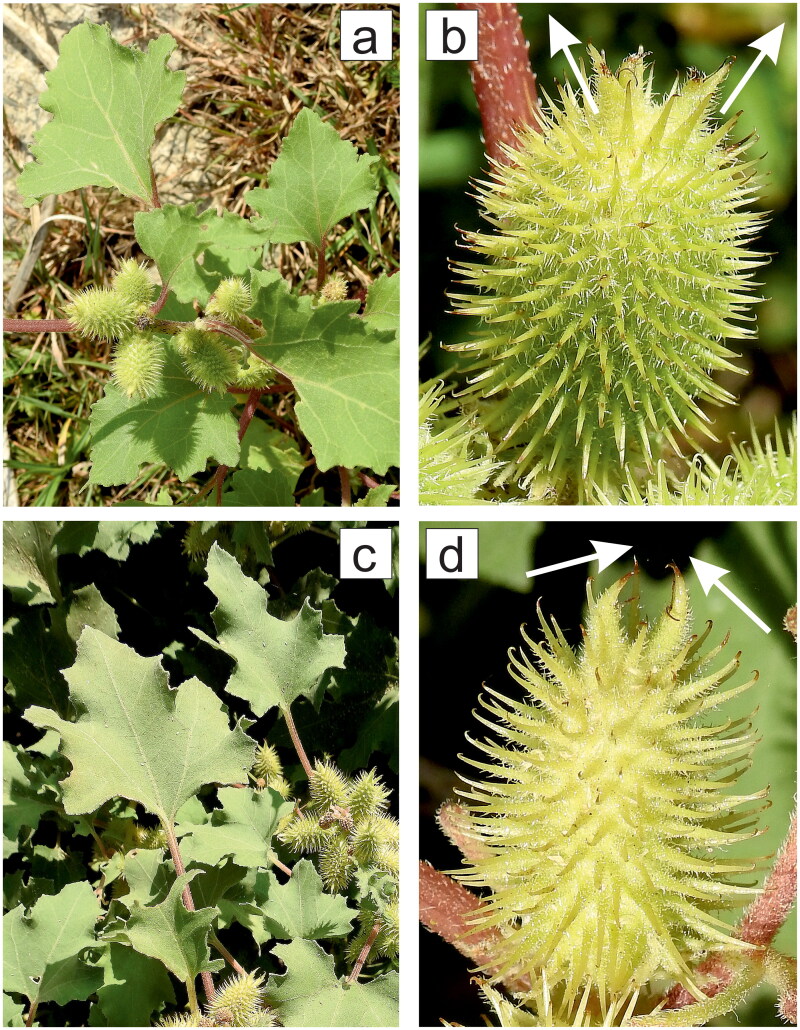
Burs and leaves of *Xanthium* spp. Regardless of the cocklebur taxon, an ovoid pod is covered with numerous stiff, hooked involucral spines and ends with two hamates, i.e., stout thorns at the apex; the edges of the leaves are irregularly serrated. Magnification image of the surface of the bur shows the details of the barbed prickles (**b** and **d**). Image of *X. strumarium* found along the Warta River, Obrzycko, Wielkopolska/Greater Poland Voivodeship, Poland (**a** and **b**). The ellipsoid pod of *X. strumarium* (**b**) is bulkier than the bur of *X. orientale* (**d**), and the two terminal strong beaks of the common cocklebur, indicated by white arrows, are divergent (**b**). Image of *X. orientale* found on a coastal beach of the Mediterranean Sea, Faliraki on the Greek Island of Rodos (**c** and **d**). The spiny capsule of *X. orientale* (**d**) is slimmer than the bur of *X. strumarium* (**b**), and the two thorns at the apex, as indicated by the white arrows, are converged and incurved (**d**). The figure was created by the author with CorelDRAW.

**Figure 3.**
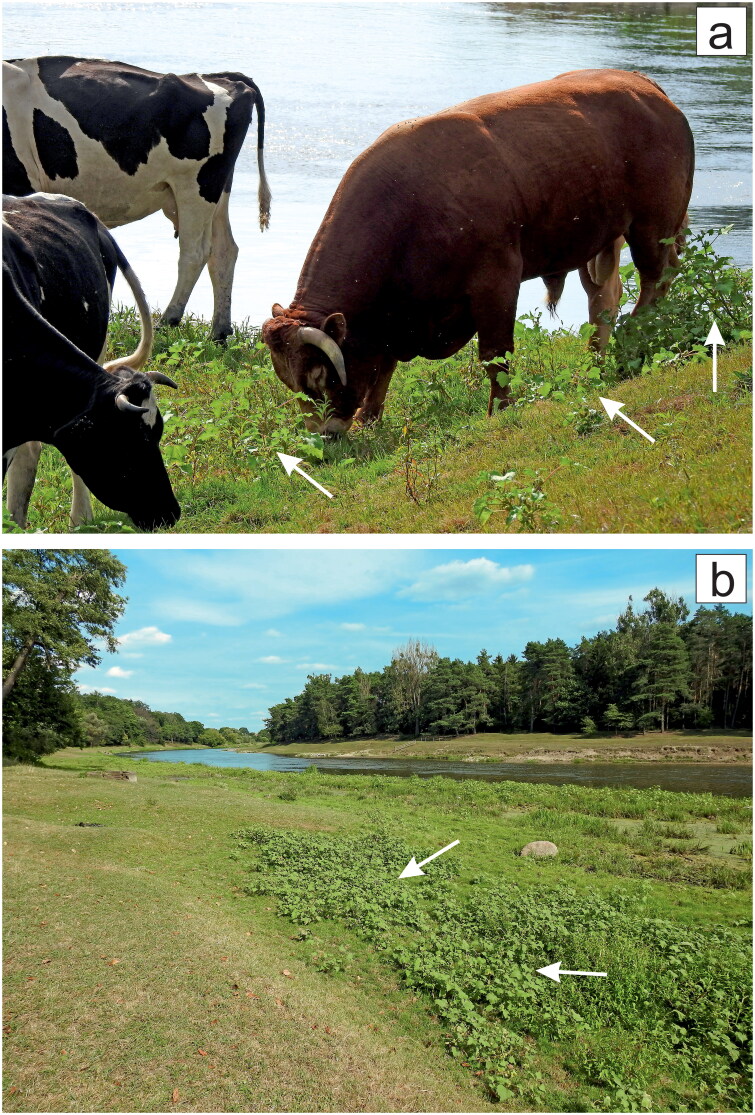
Herbivores are discouraged from eating cockleburs*. Xanthium* spp. are annual herbaceous plants with a global distribution and are considered gregarious weeds. Cattle avoid consuming free-living *X. strumarium* (**a**), while the aerial parts of other plants are almost completely consumed; see the right side of (**a**) and the left side of (**b**). White arrows indicate common cocklebur associations or single specimens near the bank of the Warta River, Obrzycko, Wielkopolska/Greater Poland Voivodeship, Poland. The figure was created by the author with CorelDRAW.

Field research related to this project led to identification of some atractyloside (ATR, Figure 1) and carboxyatractyloside (CATR, Figure 1) producers in different European locations, i.e., Poland and Greece. *Xanthium strumarium* L. (Asteraceae), which is considered a common or rough cocklebur (Figures 2(a,b) and 3), is widely distributed in Poland (Wolski et al. 2016), and *Xanthium orientale* L. (Asteraceae) (Figure 2(c,d)) belongs to the alien flora of Rodos Island (SE Aegean) (Galanos 2015). Generally, *X. strumarium* and *X. orientale* are accepted species by ‘The World Checklist of Vascular Plants' (WCVP 2022) and by ‘The World Flora Online' (WFO 2022). The stands of *Xanthium* spp. presented here are indexed for the first time. The sites of *X. strumarium* in Poland (Wielkopolska/Greater Poland Voivodeship) were located in Obrzycko Town (e.g., 52°42′29.0″N 16°30′24.7″E; initially observed on 12 August 2018 and confirmed the following summer until 2022) and Bablin Village (52°40′24.9″N 16°42′57.6″E; initially observed on 16 August 2020 and confirmed the next summer until 2022), supporting the westward expansion of common cocklebur along the Warta River. In turn, a *X. orientale* community was identified in the Faliraki resort on the Greek Island of Rodos (36°21′13.4″N 28°12′35.6″E; initially observed on 3 October 2019 and confirmed during the following autumn until 2022). To date, the available literature and databases focusing on the plant distribution/invasiveness at the local and global levels have not mentioned *Xanthium* spp. in the above-described habitats in Central and Southern Europe (see Methods). Unfortunately, the updating and precision of these databases are often neglected.

The inaccuracy of databases recording the distribution of *Xanthium* spp. may result from another issue, i.e., taxonomic chaos. For many years, the nomenclature of cockleburs has not been consistent. For example, there are many historical synonyms for *X. orientale*, such as *X. albinum* (Widder) Scholz & Sukopp, *X. canadense* Mill., and *X. strumarium* subsp. *italicum* (Moretti) D. Löve, and the synonyms for *X. strumarium* include *X. sibiricum* Widder, *X. strumarium* subsp. *sibiricum* (Widder) Greuter, *X. chinense* Mill., and *X. strumarium* var. *strumarium* L., although the recommended databases are not always convergent (WCVP 2022; WFO 2022). In particular, in December 2022, according to WFO, *X. occidentale* Bertol. is an ambiguous species name and *X. pungens* Wallr is an accepted species name, but WCVP reports these names as the synonyms of *X. chinense* (accepted species name). Moreover, WFO (2022) indexed *X. orientale* as a synonym of *X. strumarium* subsp. *strumarium* L., while WCVP (2022) indicated both *X. orientale* and *X. strumarium* subsp. *strumarium* as the accepted names of species and subspecies, respectively. This perplexing situation suggests that *X. orientale* may in fact be an ecotype/hybrid/transition morphotype of *X. strumarium*, which is supported, e.g., by internal transcribed spacer (ITS) sequencing (Noedoost et al. 2021). Therefore, the term ‘*X. strumarium* complex' might reflect a spectrum of this polymorphic species (Noedoost et al. 2021; Müller-Kiefer and Tomasello 2022). This multitude of names results from the fact that the members of the *Xanthium* genus exhibit extensive variation in mature bur morphology within the same population and between isolated populations (Figure 2), including the size of the bur, which is frequently and inaccurately referred to as fruit (Löve and Dansereau 1959; Löve 1975; Hare 1980; Weaver and Lechowicz 1983; Abbas et al. 1999; Turgut et al. 2005; Tomasello 2018; Noedoost et al. 2021; Ullah, Khan, Ali, et al. 2021; Ullah, Khan, and Rahman 2021; Jepson Flora Project 2022; Ullah, Khan, and Ali 2022; Ullah, Khan, Hewitt, et al. 2022). In fact, the bur is the fruit pod developed from fused involucre in which bract tips are transformed into spines, and the bur derives from fruiting, thus the female (pistillate) flower head (capitulum). The dissimilar morphology of cocklebur representatives worldwide may result from geographical clines, environmental effects, somatic mutations, or epigenetics (Pereira Coutinho et al. 2021). Fortunately, the number of actual taxa has decreased following revisions mainly based on genetic analyses, such as restriction fragment length polymorphism, and these studies are still ongoing to establish unambiguous species boundaries (Tomasello and Heubl 2017; Tomasello 2018; Noedoost et al. 2021; WCVP 2022; WFO 2022). However, the proposal in 1959 to simplify the *Xanthium* genus containing only two species, *X. spinosum* L., a relatively stable taxonomic entity, and *X. strumarium*, a collective name for variable biotypes, has not been commonly implemented in practice thus far, although it was supported by genetic analyses conducted decades ago and more recently (Löve and Dansereau 1959; Moran and Marshall 1978; Kelečević et al. 2021). Other modern molecular tests based on nuclear (including ITS) and plastid genetic markers circumscribed the following five taxa of the *Xanthium* genus: *X. spinosum*, *X. ambrosioides* Hook. & Arn., *X. strumarium*, *X. orientale*, and *X. chinense* (Tomasello 2018). This division is supported by an analysis of the pollen morphology of the *Xanthium* genus (Pereira Coutinho et al. 2021). Unfortunately, even in recent reports and current commercial offers, ambiguous terminology for *X. strumarium*, such as *X. sibiricum* (Jiang, Yang, Xing, Yan, Guo, Yang, et al. 2019; Somaratne et al. 2019; Sigma–Aldrich 2022), and for *X. orientale*, such as *X. cavanillesii* Schouw (Rice et al. 2019; Barbosa et al. 2020; Machado et al. 2021) or *X. italicum* Moretti (Grădilă and Jalobă 2018; Zhang et al. 2021), is used, and inconsistent terms for bur and fruit, and achene (true dry fruit) and its seed encapsulated in the bur have been used (Ghahari et al. 2017; Rice et al. 2019; Sheng et al. 2019; Sultana et al. 2019; Abdessemed et al. 2020; Amini et al. 2020; Barbosa et al. 2020; Gorbanenko 2020; Iqbal et al. 2020, 2021; Khan et al. 2020; Tong et al. 2020; Jun et al. 2021; Machado et al. 2021; Noedoost et al. 2021; Ozturk et al. 2021; Shkondrov et al. 2021; Kececi et al. 2022; Keskin Alkaç et al. 2022; Roh et al. 2022; Ullah, Khan, Hewitt, et al. 2022). Therefore, the terminology for the *Xanthium* genus and the terms for some parts of plants included in this genus must be unified as soon as possible.

The two *ent-*kaurane diterpenoid glycosides have slightly different structures, and CATR is the 4-carboxylated precursor of ATR (Figure 1) (Riccio et al. 1973; Vignais et al. 1973; Daniele et al. 2005; García et al. 2007). The original name of CATR, which is currently very rarely used, was gummiferin(e), derived from the thistle *Atractylis gummifera* L. (Asteraceae) (Vignais et al. 1971; Bouabid et al. 2019). ATR/CATR constitute, among others, the main principle of defenses for certain plants (see the following sections). Moreover, these chemical compounds are widely believed to represent extraordinary formulas provided by nature and are particularly important biochemical tools in the bioenergetics field because of a single target in mitochondria (Klingenberg 2008; Bertholet et al. 2019), but this assumption should be interpreted with caution (Table 1 and related sections).

**Table 1.** Reported direct and indirect mitochondrial targets of atractyloside and carboxyatractyloside.

**DIRECT mitochondrial targets**	**INDIRECT mitochondrial targets**
**AAC** **AAC-dependent ADP/ATP turnover** (Klingenberg 2008) **AAC-mediated H^+^ leak** (Andreyev et al. 1989; Echtay et al. 2003; Woyda-Ploszczyca and Jarmuszkiewicz 2014a)	**F_O_F_1_-ATP synthase/ATPase **(Kinne-Saffran and Kinne 1979; Ebel and Ruf 1985; Woyda-Ploszczyca and Jarmuszkiewicz 2014a)
**tricarboxylate(citrate)carrier **(Shug and Shrago 1973; Morel et al. 1974)	**TCA cycle enzymes **(Santi 1958; Xue et al. 2014)
**mtNMPK **(Allmann et al. 1967)	**UCP **(Echtay et al. 2003; Parker et al. 2008; Woyda-Ploszczyca and Jarmuszkiewicz 2014a, 2017)
**mtNDPK? **(Allmann et al. 1967)	**mtNDPK **(Woyda-Ploszczyca and Jarmuszkiewicz 2014a)

AAC: ADP/ATP carrier; mtNDPK: mitochondrial nucleoside diphosphate kinase; mtNMPK: mitochondrial nucleoside monophosphate kinase; TCA cycle: tricarboxylic acid cycle; UCP: uncoupling protein; ?: possibly direct inhibition. Representative references are cited.

The therapeutic properties of plants synthesizing ATR/CATR have been known for a long time. *A. gummifera* was used in ancient Greece and throughout most of the Mediterranean Basin (Daniele et al. 2005; Bouabid et al. 2019). Among the Zulu and Xhosa people of South Africa, the suffrutext *Callilepis laureola* DC. (Asteraceae) has had curative value (Stewart and Steenkamp 2000; Steenkamp et al. 2004; Brown 2017). From early China and the pre-Chinese period, even ca. the 45^th^–40^th^ centuries BC (the Neolithic period), *X. strumarium* (Figures 2(a,b) and 3) has probably been among the major sources of herbal medications in Asia (Fan et al. 2019; Sheng et al. 2019; Sultana et al. 2019; Khan et al. 2020). There are also reports indicating the use of *X. strumarium* for medicinal purposes by native inhabitants of America (Sheng et al. 2019; Lawson et al. 2020). However, the toxic and healing effects of these plants are still not completely understood. The complex interrelationships between ATR/CATR and their target proteins, both direct and indirect, including those forgotten and not considered to date, are the final subtopic of this review (Table 1 and related sections). The extended affinity of the cell for these glycosides through different enzymes/carrier proteins suggests that this aspect must be extensively investigated and, thus, urgently revised and universally updated.

## Methods

The nomenclature of *Xanthium* spp. is based on ‘The World Checklist of Vascular Plants, Royal Botanic Gardens, Kew' (WCVP 2022) in partnership with the ‘Global Biodiversity Information Facility (GBIF)' (Roy et al. 2020), and ‘The World Flora Online' (WFO 2022). The local/global presence and distribution of *Xanthium* spp. were determined using the following databases: the ‘Alien Species in Poland' (pol. *Gatunki Obce w Polsce*) (Gatunki Obce w Polsce 2022), the ‘CABI (Centre for Agriculture and Bioscience International) - Invasive Species Compendium' (CABI 2022), the ‘DAISIE (Delivering Alien Invasive Species Inventories for Europe) - Inventory of alien invasive species in Europe' in partnership with the ‘GBIF' (Roy et al. 2020), the ‘Alien Plants in Greece: a web-based platform' (Alien Plants in Greece 2022), and the ‘Flora of Greece Web' (Flora of Greece Web 2022). Information concerning the potential toxicity of *Xanthium* spp. was searched in ‘European Food Safety Authority' (European Food Safety Authority 2012) and ‘FDA (United States Food and Drug Administration) - Poisonous Plant Database' (FDA 2022). The literature and other data were searched in ACS Publications, Acta Scientiae Veterinariae, BioOne Complete, Canadian Science Publishing, Google/Google Scholar, Oxford Academic, Postępy Fitoterapii, PubMed, ResearchGate, RSC Publishing, SciELO Brazil, ScienceDirect, Sciendo, SpringerLink, Taylor & Francis Online, TÜBİTAK Academic Journals, and Wiley Online Library databases between 1957 and December 2022 using the following keywords: ‘ADP/ATP carrier (AAC)', ‘atractyloside', ‘carboxyatractyloside', ‘cattle', ‘cockleburs', ‘hepatorenal syndrome', ‘hepatotoxicity', ‘mitochondria', ‘molecular interactions', ‘nephrotoxicity', ‘nucleoside diphosphate kinase (NDPK)', ‘oxidative phosphorylation', ‘proton (H^+^) leak', ‘Traditional Chinese Medicine', ‘uncoupling protein (UCP)', and *‘Xanthium*'.

## Pharmacological and other applications of plants producing atractyloside and carboxyatractyloside

Uniquely, the oldest fossil evidence of common cocklebur seeds found in Europe to date has been discovered in central Poland (Mueller-Bieniek et al. 2015). Intriguingly, the *X. strumarium* relics colocalized with a human-made tool, i.e., a stone grinder, other medicinal plants, and crops. These charred macroremains, which were probably processed by ancient people, were radiocarbon dated to the Late Bronze Age, ca. the 10^th^–8^th^ centuries BC. Thus, representatives of primeval Lusatian culture who historically settled mainly in the region of modern Poland may have benefited from ancient Chinese medical experience, currently termed Traditional Chinese Medicine (TCM). The land corridor from the far east to Central and Western Europe might run through the territory of current-day Ukraine, where some archaeological sites of human settlements were marked by burs of *X. strumarium* (Gorbanenko 2020). This biological material, since at least the beginning of the Early Iron Age, e.g., dated to the second half of the seventh century BC, is presumed to have been deliberately picked up. Therefore, Mediterranean cultures were not necessarily the first users of *Xanthium*-based medicine in Europe (Müller-Kiefer and Tomasello 2022), although the supposition regarding the Mediterranean-European or American origin of *X. strumarium* is still repeated in various sources (Iqbal et al. 2020, 2021; Kelečević et al. 2020; Saeed et al. 2020; Machado et al. 2021; Shkondrov et al. 2021; Ullah, Khan, Ali, et al. 2021; Ullah, Khan, and Rahman 2021; CABI 2022; Jepson Flora Project 2022; Ullah, Khan, and Ali 2022; Ullah, Khan, Hewitt, et al. 2022; WCVP 2022; WFO 2022). Alternatively, the use of *X. strumarium* as a medicine could have been invented by our ancestors independently/simultaneously in Europe, America, and Asia. The real origin of this plant is uncertain and should be reconsidered, and the Far East has also been contemplated, suggesting that *X. strumarium* could be a species native to Eurasia (CABI 2022; WCVP 2022).

Independent extracts of *A. gummifera*, *C. laureola* and *X. strumarium*, often supplemented with other ingredients, have been exploited for medicinal purposes, including curing rhinitis, allergic catarrh, and related nasal ailments, such as sinusitis (Fan et al. 2019; Sheng et al. 2019; Sultana et al. 2019; Khan et al. 2020); combating headache; as a vermicide against intestinal worms, e.g., tapeworm (Steenkamp et al. 1999; Stewart and Steenkamp 2000; Daniele et al. 2005); as a remedy for fungal infections (Fan et al. 2019; Khan et al. 2020), leprosy (Daniele et al. 2005) and leukoderma, e.g., a consequence of psoriasis (Fan et al. 2019; Khan et al. 2020; and references therein). Modern *in vitro* and *in vivo* studies have disclosed that *Xanthium*-derived compounds/preparations may possess broad-ranging pharmacological activities, including antitumor (e.g., anti‑breast cancer and antileukemia), antidiabetic, antiblood parasite (e.g., plasmodicidal), antibacterial (e.g., toward *Staphylococcus epidermidis*), and antiviral (e.g., against influenza A virus) properties; thus, the cocklebur genus has promising prospects for the identification of effective pharmaceuticals (Kamboj and Saluja 2010; Al-Mekhlafi et al. 2017; Fan et al. 2019; Sultana et al. 2019; Khan et al. 2020; and references therein). Moreover, the insecticidal and repellent actions of *Xanthium*-based mixtures have been reported. Innovatively, the seed oil of cocklebur, which is considered nonedible and whose content may be over 42% (wt/wt), has been suggested to serve as a biofuel (Chang et al. 2013; Rozina et al. 2017; Cesur et al. 2018). This oil is rather uncontaminated with ATR/CATR, which are polar, hydrophilic compounds with relatively poor solubility in organic solvents (Vignais et al. 1971; Cole et al. 1980; Cutler and Cole 1983; Obatomi and Bach 1998; Steenkamp et al. 2004; Yang et al. 2013; Xue et al. 2014; Nikles et al. 2015; Shkondrov et al. 2021). *Xanthium* leaf or seed oil has also been declared another treatment ‘tool' for diseases, such as herpes (Kamboj and Saluja 2010; Sultana et al. 2019; Khan et al. 2020).

## Toxicity of atractyloside- and carboxyatractyloside-containing plants with a focus on *Xanthium* spp.

Historical experiences are often translated to the contemporary world, particularly in areas in which ethnopharmacology, including veterinary folk medicine, is still practiced, and plays an influential role in society. Unfortunately, the risk of toxicosis has been noted when using ATR/CATR producers. *A. gummifera* and *Xanthium* spp. are recorded as potentially dangerous by the United States Food and Drug Administration (FDA) in the Poisonous Plant Database (FDA 2022) and the European Food Safety Authority (European Food Safety Authority 2012). Similarly, *C. laureola* is indexed in books concerning toxic and injurious Southern African plants (Stewart and Steenkamp 2000) and the FDA (2022). Concerns regarding traditional herbal medicines are increasing as a result of patients developing noxious side effects due to an immoderate intake of crude and noncontrolled remedies who must then be urgently hospitalized. For many years, alternative treatments have become more popular, which may pose a serious threat to people without adequate knowledge, e.g., people from metropolitan areas (Stewart and Steenkamp 2000). Importantly, the administration of decoctions or infusions from ATR/CATR-containing plants or the accidental ingestion of these plants may lead to fatality in humans, including death within a short time after consumption (Georgiou et al. 1988; Obatomi and Bach 1998; Stewart and Steenkamp 2000; Daniele et al. 2005; Turgut et al. 2005; Nya et al. 2021). Intriguingly, *in vitro* research using human liver and kidney cell lines and *in vivo* experiments using rodents indicate that chronic overdosing may be critical for the high cytotoxicity and death caused by *Xanthium*-derived extracts (Yu et al. 2013; Schiller et al. 2017). Even more worrisome, these findings are supported by incidents recorded in low-income regions with increased famine due to extreme weather conditions. In 2007, an outbreak of complications followed by high mortality (25%; 19 deaths among 76 patients), predominantly in children aged ≤ 15 years, occurred following the long-term consumption of uncultivated seedlings, most of which were shown to be *X. strumarium* (Gurley et al. 2010). This situation resulted from inaccessibility to other food resources after destructive monsoon floods in Bangladesh, where crop loss ultimately caused a rapid increase in grocery prices.

The toxicity of all cockleburs appears to be similar (Stuart et al. 1981). Strikingly, *Xanthium* spp. sprouts, i.e., the assimilating cotyledon stage with two linear lanceolate leaf-like structures, which do not resemble the true leaves, are recorded as the predominant agent of poisoning (Rostafiński and Seidl 1973; Cole et al. 1980; Stuart et al. 1981; Scherer et al. 2009; Gurley et al. 2010; Botha et al. 2014; Rice et al. 2019; Barbosa et al. 2020; Machado et al. 2021; Ullah, Khan, Hewitt, et al. 2022). A possible explanation is that CATR is not transiently detectable in older plants without burs containing achenes, i.e., from the four-leaf stage onward (Cole et al. 1980). However, cockleburs at the four-leaf stage with attached cotyledons are still toxic to mammals (Mendez et al. 1998). A comparative study revealed that among the different dried plant parts of *X. strumarium*, cotyledonary leaves contained the highest CATR concentration, i.e., 48.6 μg/g, while only approximately 1/35^th^ of this concentration was detected in unbroken burs, i.e., containing achenes, which were untouched (not ingested by cattle), namely, 1.4 μg/g (Botha et al. 2014). However, a recent analysis reported much higher concentration values in *X. strumarium* seedlings, i.e., 300 μg/g ATR and 370 μg/g CATR, and in intact burs, i.e., 22 μg/g ATR and 3.7 μg/g CATR, from dried plant material (Machado et al. 2021). Moreover, mature leaves of *X. strumarium* incorporated almost no CATR, i.e., < 0.01 μg/g (Botha et al. 2014), and no ATR (Ozturk et al. 2021). Indeed, CATR may be undetectable in fully grown leaves and mature spiny involucres (burs without true dry fruits), but achenes, which are very often mistakenly referred to as seeds, enclosing a single kernel and cotyledonary-stage leaves are the source of this toxin in *X. strumarium* (Scherer et al. 2009). A complicating finding of an earlier *X. strumarium* analysis was the presence of CATR in integral burs at a significantly higher concentration, i.e., 4.57 mg/g, than that detected in very young seedlings at the two leaf-like phase, i.e., 1.2 mg/g (Cole et al. 1980). The extraction recovery of approximately 4.6 mg/g CATR in complete *X. strumarium* burs was supported by an independent analysis (Witte et al. 1990). Gently dehydrated whole burs of *X. strumarium* may contain much more CATR than ATR (Nikles et al. 2015), but ATR is still present at levels above trace amounts in these organs, sometimes reaching ca. 2.57 mg/g (Chen et al. 2013). Principally, the uncut burs of *X. strumarium* have been pulverized for ATR/CATR analyses (Cole et al. 1980; Witte et al. 1990; Chen et al. 2013; Botha et al. 2014; Nikles et al. 2015; Machado et al. 2021), but achenes may constitute ca. 25% (wt/wt) of this structure at a minimum and even 38% (wt/wt) on average (Weaver and Lechowicz 1983; Witte et al. 1990). Therefore, the ATR/CATR content detected in the undivided bur may actually constitute the whole pool present in the true fruits, mainly in seeds. Interestingly, CATR has been reported to be concentrated in the spines of *Xanthium* burs (Figure 2) (Kamboj and Saluja 2010; Tomasello and Heubl 2017). However, according to a precise analysis of common cockleburs, the core molecule of CATR, i.e., ATR, is stored mainly in the seed (defined by the authors as seed kernel) at a concentration of up to 7.917 mg/g; therefore, the prickles, which may have an ATR content of up to 0.1612 mg/g, are not the reservoir of these toxic glycosides (Yang et al. 2013). In the mature involucre without bristles (defined by the authors as the shell of the bur) and the pericarp (probably ascribed to the seed coat by mistake), concentrations of up to 0.1427 and 0.3888 mg/g ATR, respectively, were detected. These findings were based on an analysis of *Fructus xanthii*, i.e., ripe, intact (with achenes), dried burs of *X. strumarium*, in which some probes were processed *via* stir baking or collected even a few years earlier before laboratory tests. Nevertheless, separate analyses revealed a total lack of ATR and CATR in the spikes of this type of *Fructus xanthii* (gently dried) (Nikles et al. 2015). This result is consistent with a previous study showing the absence of CATR in the spiny burs of *X. strumarium* from which the achenes were removed (Scherer et al. 2009). In fresher achenes (probably defined by the authors as seeds by mistake), i.e., harvested in the year of fruiting, the mean ATR concentration depended on the month during which the samples were collected and was calculated as 3.043 mg/g in the samples collected in August, 3.502 mg/g in the samples collected in September, and 3.8 mg/g in the samples collected in October (Ozturk et al. 2021). Generally, a concentration of 4 mg/g ATR in new generation achenes collected in autumn (e.g., in October), thus from freshly dried burs of *X. strumarium*, was also reported (Kececi et al. 2022; Keskin Alkaç et al. 2022). Moreover, mature (May–October) roots, stems, and leaves of common cockleburs lack ATR (Ozturk et al. 2021). Therefore, only the adult foliage and stalks of *X. strumarium* are adequate for medicinal or culinary purposes, e.g., to flavor curries (Scherer et al. 2009; Gurley et al. 2010).

Similar to animals and humans, plants are also sensitive to ATR/CATR (Vignais et al. 1976). Based on an analysis of the tubers of *C. laureola* at a subcellular level, these glycosides are predominantly stored in vacuoles to avoid autointoxication of the host (Dehrmann et al. 1991). The exact mechanism of the sequestration of different plant toxins in sites, such as central vacuoles, has not been fully explored (Brandle and Telmer 2007; de Brito Francisco and Martinoia 2018).

## *Xanthium* spp. as a problem for livestock

Livestock are also exposed to cocklebur poisoning, particularly when cattle or swine are allowed to graze on wild pasture land (Cole et al. 1980; Stuart et al. 1981; Botha et al. 2014; García et al. 2017; Rice et al. 2019; Barbosa et al. 2020; Jun et al. 2021; Machado et al. 2021). Adverse effects have been reported after plant ingestion, including fatalities, i.e., 6 deaths per 70 individuals, in yearling beef calves unwisely fed round bale hay consisting largely of *Setaria* spp. (foxtail grasses) and mature *X. strumarium*; burs constituted 30% (wt/wt) of this feedstuff (Witte et al. 1990). Whole and crushed burs of *Xanthium* spp. are often present in forage, e.g., silage, as these plants may grow among crops, which are usually harvested without discarding cocklebur contamination (Goodwin et al. 1992; Jun et al. 2021; Roh et al. 2022). Impressively, a single representative of *X. strumarium* may produce thousands of burs under proper conditions, i.e., up to 5400 (Weaver and Lechowicz 1983). In contrast to cattle, in broiler chickens intentionally fed a 25% (wt/wt) cocklebur diet, the only effect was a substantial decrease in the body weight of the cohorts by over half on average after 21 days (Goodwin et al. 1992). The weight loss was attributed to either the low nutritional value of this type of fodder or a specific response to prevent CATR intoxication at a lethal level in young poultry. In cattle, fatal intoxication with *Xanthium* spp. among the herd is often relatively rare, e.g., 4 deaths per 150 individuals, and mortality was generally estimated to range from 2 to 5.5% but may be much higher and even reach 58% (Mendez et al. 1998; Botha et al. 2014; García et al. 2017; Rice et al. 2019; Barbosa et al. 2020; Machado et al. 2021). In my original field observations (see Introduction) of bulls, cows and calves engaged in free-range grazing on a riverbank (initially on 12 August 2018), all farm animals avoided the consumption of mature common cocklebur ‘weeds' in Poland (Figure 3). This natural instinct, regardless of age, may represent a type of behavioral imprinting. Presumptively, both the high concentration of ATR/CATR in seeds and the rough texture of *Xanthium* spp. renders adult plants of this genus nonpalatable to most herbivores (Weaver and Lechowicz 1983; Goodwin et al. 1992; Turgut et al. 2005). The relatively old Polish botanical key also indicates that livestock do not usually eat cockleburs (Rostafiński and Seidl 1973). However, immature *Xanthium* cohorts, e.g., seedlings in the ground, may not be recognized by cattle of various ages (Rice et al. 2019; Machado et al. 2021) or humans (Gurley et al. 2010). A similar situation may apply to fully grown cockleburs (Botha et al. 2014; Barbosa et al. 2020).

## Basics of atractyloside and carboxyatractyloside toxicities and potential antidotes

The glycosides ATR and CATR are the main components inducing acute death in the abovementioned examples. Nevertheless, ATR/CATR toxicosis may be partially derived from the *de novo* synthesis of some reactive metabolites *in vivo* after biotransformation (Hatch et al. 1982; Krejci and Koechel 1992; Koechel and Krejci 1993; Yu et al. 2013). These xenobiotics are cytotoxic and primarily cause hepatotoxicity and nephrotoxicity, which has been explicitly indicated in humans, domesticated and laboratory animals, and different mammalian cell lines (Cole et al. 1980; Stuart et al. 1981; Georgiou et al. 1988; Witte et al. 1990; Krejci and Koechel 1992; Koechel and Krejci 1993; Roeder et al. 1994; Obatomi and Bach 1996a, 1998; Mendez et al. 1998; Obatomi et al. 1998, 2001; Stewart and Steenkamp 2000; Daniele et al. 2005; Turgut et al. 2005; Yin et al. 2008; Hamouda et al. 2010; Wang et al. 2011; Yu et al. 2013; Botha et al. 2014; Xue et al. 2014; Fan et al. 2019; Rice et al. 2019; Barbosa et al. 2020; Jun et al. 2021; Machado et al. 2021; Keskin Alkaç et al. 2022; Roh et al. 2022). Moreover, hepatonephrotoxicity may be accompanied by multiple organ dysfunction involving myocardial and lung injury (Turgut et al. 2005; Roh et al. 2022). Another analog of ATR, 4′-desulfate-atractyloside, extracted from the utter burs of *X. strumarium*, also has hepatotoxic potential (Xue et al. 2014). As noted above, ATR/CATR are polar (anionic) and hydrophilic; thus, the extent of passive (direct) diffusion through the phospholipid bilayer is rather negligible for these generally impermeant molecules (Klingenberg et al. 1971; Vignais et al. 1973). Nevertheless, these glycosides are postulated to possess amphiphilic properties (Vignais 1976; Brandolin et al. 1980). Researchers have hypothesized that some regioselective organic ion transport systems (carrier protein-dependent), expressed in renal proximal tubule cells, participate in the traversal of the cell membrane by ATR/CATR (Krejci and Koechel 1992; Koechel and Krejci 1993; Obatomi and Bach 1996b; Obatomi et al. 1998, 2001; Daniele et al. 2005; Turgut et al. 2005). Other cell types devoid of special transporters may be less susceptible to the immediate exposure to these glycosides. For example, striated muscles in vertebrates may be resistant to ATR and, thus, are nontarget tissues (Obatomi et al. 1998).

**Figure 4.**
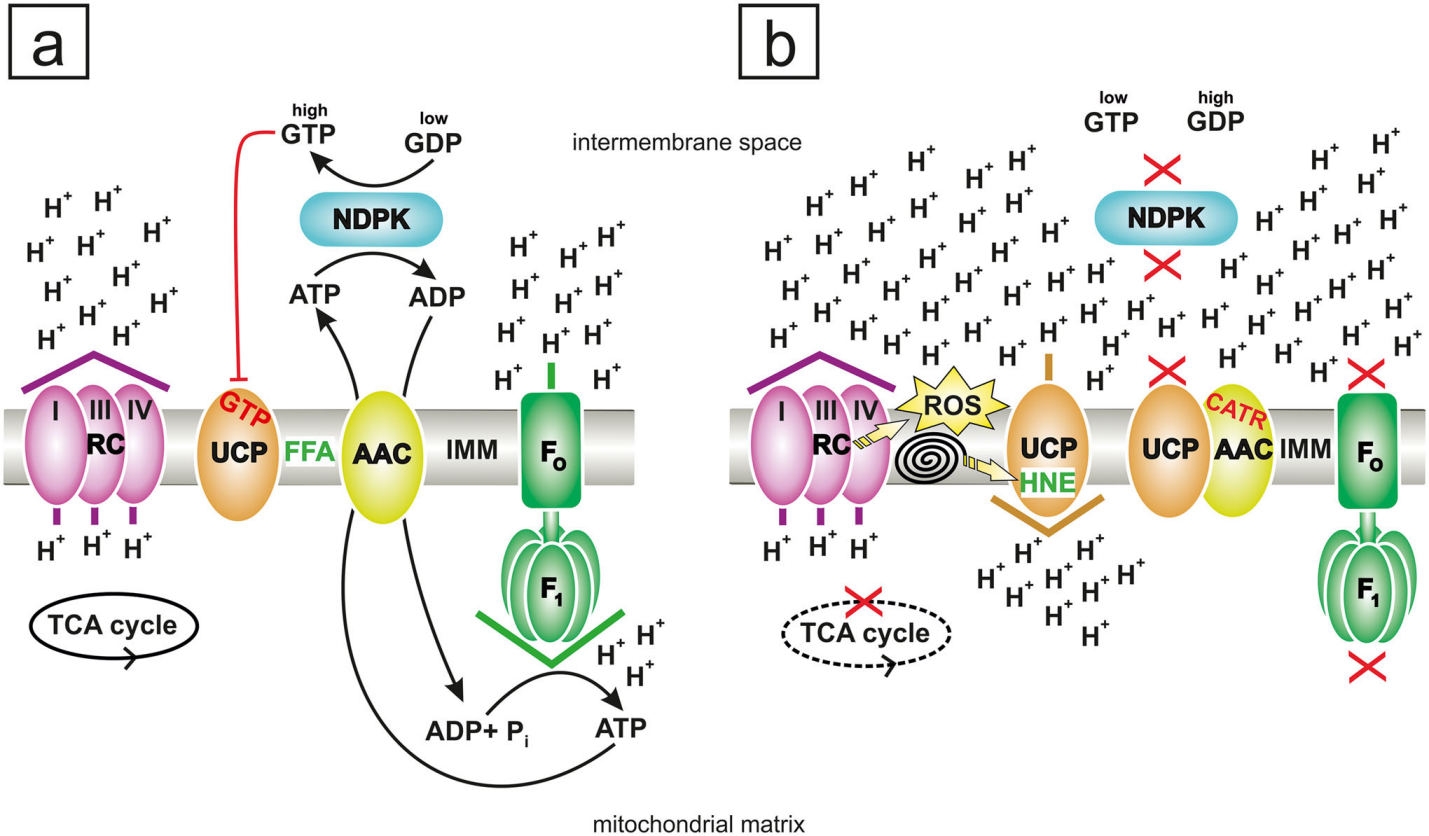
The extended effect of carboxyatractyloside (CATR) on mitochondria. (**a**) In the absence of CATR, the ADP/ATP carrier (AAC) is primarily employed as an antiporter of nucleotide metabolites, although free fatty acids (FFAs), which are prospective activators of H^+^ leak *via* AAC and uncoupling protein (UCP), are present in the inner mitochondrial membrane (IMM). Efficient active mitochondrial nucleoside diphosphate kinase (NDPK) continuously generates ADP and GTP using ATP and GDP as substrates *via* the 'ping-pong' mechanism. The ADP pool induces oxidative phosphorylation (OXPHOS), which translates into a low H^+^ gradient, originally generated by the respiratory chain (RC), due to the effect of its consumption by F_O_F_1_-ATP synthase (F_O_F_1_). Although the access of UCP to GDP is limited, this transporter is quite strongly inhibited. First, a physiological need for mild uncoupling does not exist under OXPHOS conditions. Second, the NDPK-mediated transphosphorylation of GDP produces GTP, i.e., the weaker negative regulator of UCP is substituted with the stronger inhibitor in the intermembrane space of the mitochondria toward which UCP exposes the PN-binding site. The indirect exclusion of UCP activity by NDPK, residing in the intermembrane space, does not cause net energy losses in cells. Specifically, NDPK consumes ATP but delivers precious GTP and additionally rescues the H^+^ gradient from dissipation *via* UCP. AAC-perpetrated mitochondrial H^+^ conductance is not shown for clarity and due to its minor physiological significance during OXPHOS. In mammals and possibly many other organisms, NDPK is bound electrostatically to cytosolic-facing and matrix-facing leaflets of the IMM, but for simplicity, the schematic transphosphorylation reaction is shown only in the intermembrane space and outside of the IMM. The phosphoenzyme intermediate of NDPK is also omitted. (**b**) CATR intoxication. When ADP does not enter the mitochondrial matrix because CATR binds to AAC, the electrochemical H^+^ gradient is much higher than that in the model shown in (**a**). This outcome is due to the almost complete but indirect inhibition of F_O_F_1_-ATP synthase by CATR. This situation creates perfect conditions to initiate uncoupling, which is facilitated and driven by a high H^+^ gradient. UCP is not necessarily strongly inhibited; although the GDP concentration probably increases, this nucleotide apparently shows lower specificity for UCP, and GTP (considered the physiologically relevant inhibitor of UCP) deficiency occurs. GTP is not formed abundantly because NDPK, such as F_O_F_1_-ATP synthase, is indirectly quenched by CATR, which limits the access of the kinase to ATP. Moreover, the overreduction of RC favors increased reactive oxygen species (ROS) production and subsequent lipid peroxidation, which is depicted as a 'spiral'. In turn, the reactive aldehyde 4-hydroxy-2-nonenal (HNE), one of the most abundant secondary lipid peroxidation end products, becomes an activator of AAC/UCP-dependent H^+^ leak, which may be partially reversed by CATR. Potential AAC-UCP heterodimers may feasibly be restrained by CATR, which excludes the full contribution of each carrier protein component to H^+^ conductance. The blockade of AAC-UCP heterodimers by CATR additionally exacerbates the metabolic crisis as it eliminates the prominent element of first-line antioxidant defenses. Some rescue may be achieved by UCP that is not complexed with AAC, which might be involved in the HNE-stimulated pathway of H^+^ leak. Thus, mainly/only AAC-free UCP counteracts CATR-induced oxidative stress by catalyzing a low degree of uncoupling. However, UCP-driven 'futile' H^+^ passage across the IMM may postpone/minimize the adverse symptoms of CATR poisoning. Importantly, the scale of beneficial antioxidative H^+^ leak depends on the relative concentrations of effectors implicated in the promotion and inhibition of AAC/UCP-dependent uncoupling. Finally, the substantial ATR/CATR-dependent attenuation of ATP regeneration *via* OXPHOS during acute intoxication negatively and irreversibly affects organ structures that are highly sensitive to these glycosides, such as the kidney and liver, often leading to the death of humans and animals. The direction of H^+^ movement against or down the electrochemical gradient is indicated by the colored arrowhead placed above or below a particular protein component of the IMM, and violet arrowhead represents RC activity (**a** and **b**), brown represents UCP activity (**b**), and green represents F_O_F_1_-ATP synthase activity (**a**). The red 'X' (**b**) indicates indirect inhibition elicited by CATR, and the dashed black ellipse (**b**), symbolizing the tricarboxylic acid (TCA) cycle, corresponds to the slowing of its rate. I, III and IV: three H^+^-pumping complexes of RC. P_i_: inorganic phosphate. The figure was created by the author with CorelDRAW.

The ATR and CATR are very toxic hypoglycemic agents (Kupiecki et al. 1974; Craig et al. 1976; Stuart et al. 1981; Krejci and Koechel 1992; Koechel and Krejci 1993; Stewart and Steenkamp 2000; Jun et al. 2021; Keskin Alkaç et al. 2022). This finding is not surprising as the two glycosides are responsible for impairing mitochondrial oxidative phosphorylation (OXPHOS). Specifically, ATR/CATR are direct inhibitors of ADP/ATP carrier (AAC) proteins and block cellular ADP/ATP cycling by binding to the translocase from the cytosolic side (Figure 4(b) and Table 1) (Vignais 1976; Klingenberg 2008); AACs are embedded in the inner mitochondrial membrane (IMM) (Figure 4). Hence, severely disordered mitochondrial morphology, e.g., degeneration by swelling and blockade of cell division, is caused by ATR and CATR (Stewart and Steenkamp 2000; Machado et al. 2021). The two glycosides are similar to adenine nucleotides in geometry, size, and charge distribution (Allmann et al. 1967; Stewart and Steenkamp 2000; Obatomi et al. 2001; Klingenberg 2008). Moreover, the structures of ATR/CATR are similar to that of oligomycin (Figure 1), an antibiotic that hampers OXPHOS but directly inhibits F_O_F_1_-ATP synthase/ATPase (Figure 4) (Al Maruf et al. 2014). In the absence of ADP entry into mitochondria to renew ATP, the oxidative reactions of the tricarboxylic acid (TCA) cycle are also slowed down/paused, and thus, this cycle is indirectly attenuated/inhibited by ATR/CATR (Figure 4(b) and Table 1) (Santi 1958; Xue et al. 2014). Consequently, a conspicuous hyperglycemia phase driven by the rapid depletion of glycogen deposits from skeletal muscle and the liver is usually an early symptom of ATR/CATR poisoning (Krejci and Koechel 1992; Koechel and Krejci 1993; Obatomi and Bach 1998; Turgut et al. 2005; Yu et al. 2013). As the role of anaerobic glycolysis increases, a marked hypoglycemia stage arises, usually within a few hours after ATR/CATR intake. Accordingly, both ATR/CATR exposure and AAC protein deficiency, e.g., in Sengers syndrome, a rare autosomal recessive mitochondrial disease, may elicit conditions, such as lactic or general acidosis, as a result of mitochondrial failure (Warnette-Hammond and Lardy 1985; Krejci and Koechel 1992; Koechel and Krejci 1993; Graham et al. 1997; Jordens et al. 2002; Turgut et al. 2005; Xue et al. 2014).

The biochemical basis of the cytotoxicity of both *ent-*kaurane glycosides is also mediated by an oxidative stress-induced increase in membrane lipid peroxidation, as manifested in an increased content of malondialdehyde (Obatomi and Bach 1996a; Obatomi et al. 1998; Wang et al. 2011; Keskin Alkaç et al. 2022). These data are consistent with independent studies showing that palmitoyl-coenzyme A (P-CoA), another direct negative regulator of AAC, increases reactive oxygen species (ROS) release (Ciapaite et al. 2006; Ludzki et al. 2015). Therefore, AAC inhibitors might cause sequential increases in ROS, 4-hydroxy-2-nonenal (HNE), and protein carbonylation levels under certain conditions (Ludzki et al. 2015). The aldehydes malondialdehyde and HNE, the most abundant secondary lipid peroxidation end products, are mutagenic to DNA and inhibit some proteins by covalent binding to these biopolymers (Ayala et al. 2014; Faisal et al. 2017; Rebollido-Rios et al. 2020). Finally, the membrane permeability transition (MPT) in mitochondria is promoted by ATR/CATR, including *X. strumarium* seed extract, because these glycosides induce the opening of nonspecific pores through AAC immobilization and potentially trigger apoptosis as a consequence of cytochrome *c* release from this organelle (Hernández-Esquivel et al. 2011; Keskin Alkaç et al. 2022).

Notably, CATR is much more poisonous than ATR overall (Luciani et al. 1971; Scherer et al. 1973; Vignais et al. 1976; Daniele et al. 2005; Klingenberg 2008; Wang et al. 2011; Nikles et al. 2015; Bouabid et al. 2019). For instance, the inhibitory effect of CATR is at least ten-fold stronger than that of ATR on mitochondria isolated from rat livers (Luciani et al. 1971), but the former may be forty- to fifty-times more potent in both isolated mitochondria, e.g., from potatoes (Vignais et al. 1976), and whole organisms, such as rats (Nikles et al. 2015; Bouabid et al. 2019). The CATR-dependent inhibitory effect on AAC in mammals is almost completely uncompetitive; in relation to ATR, one more negative charge centered on the CATR molecule may cause a higher binding affinity and result in quasi-irreversible action (Luciani et al. 1971; Vignais et al. 1971; Vignais 1976; Klingenberg 2008). In fact, the extra carboxylate present in CATR (Figure 1) forms a salt bridge with a specific residue of α helix 2 in AAC (Sanchez et al. 2012). Consequently, the strength of the interaction between CATR and the AAC ligand binding pocket is increased in various organisms; thus, the degree of the kinetic stabilization of this carrier protein by CATR is greater than that of ATR (Klingenberg et al. 1978; Kedrov et al. 2010; Sanchez et al. 2012). However, the stereo-isomer epi-ATR, which has an equatorial COOH group, similarly to CATR (Figure 1), also shares a comparably high affinity for AAC with CATR (Riccio et al. 1973; Scherer et al. 1973). Therefore, both the number and stereochemistry of the charged groups must determine the toxicity of these glycosides. In turn, the conventional ATR-mediated inhibition of AAC is quite effectively abrogated by molecules, such as ADP (almost completely in some cases), ATP, or P-CoA (ca. half effective), at sufficiently high concentrations based on *in vitro* studies, but an antidote for severe ATR poisoning has not yet been developed (Bruni et al. 1965; Allmann et al. 1967; Luciani et al. 1971; Vignais et al. 1971; Woldegiorgis and Shrago 1979; Obatomi and Bach 1996b, 1998; Stewart and Steenkamp 2000; Obatomi et al. 2001; Daniele et al. 2005; Ozturk et al. 2021; Kececi et al. 2022; Keskin Alkaç et al. 2022).

Among the potential antidotes, at least one candidate was viewed with optimism, i.e., probenecid, a competitive blocker of foreign organic anion uptake in kidneys; unfortunately, this compound may aggravate the injurious effect of ATR (Koechel and Krejci 1993; Obatomi et al. 2001). Probenecid likely decreases the clearance of ATR from the body *via* the kidneys and finally urine. Similarly, the selective transport of ATR across the cell membrane may be partially blocked by a plant-derived analog of ATR, stevioside (Ishii and Bracht 1986; Obatomi et al. 2001). Amazingly, this glycoside is used as a sweetener as it is up to 400-times sweeter than sucrose and is relatively safe for health, serving even as the basis of anti-HIV agents (European Food Safety Authority 2010; Kobayashi et al. 2018; Ciriminna et al. 2019). Unfortunately, stevioside, such as ATR and CATR (purified or in *Xanthium-*derived extracts), may also directly/indirectly disrupt some cellular processes and cause a significant decrease in the ATP levels (Obatomi et al. 2001; Jiang, Yang, Xing, Yan, Guo, Hou, et al. 2019; Machado et al. 2021).

Additionally, tamoxifen, a selective estrogen receptor modulator administered in breast cancer therapy that functions as an antagonist, is also of interest (Hernández-Esquivel et al. 2011). This drug nonspecifically inhibits the opening of mitochondrial transmembrane nanopores, i.e., MPT, induced by CATR, as detected in preparations of mitochondria isolated from the rat kidney cortex. Nevertheless, the protective effect of tamoxifen on decreasing membrane fluidity could be unrelated to normal human body temperature because it is gradually attenuated with increasing temperature from 25°C to 35°C. However, the antioxidant action of tamoxifen, which is also capable of preventing the binding of some undesirable ligands to AAC, such as agaric acid, might be beneficial for mammals exposed to different toxicants (Chávez et al. 2020).

The next option is calpain inhibitor I, a synthetic neutral protease blocker with a potential (partial) protective effect against ATR toxicity, as revealed *in vitro* in rat renal cortical slices (Obatomi et al. 2001). The action of this inhibitor is not fully understood but probably counteracts subcellular proteolysis and subsequent cell damage or even cell death resulting from the ATR-dependent release of some peptidases, such as lysosomal cathepsin B (Vancompernolle et al. 1998).

Researchers have also expressed some optimism concerning the use of glycyrrhizic acid, a bioactive triterpene glycoside in licorice, which is a powerful antihepatotoxic protectant against *Fructus xanthii* seed extract-induced (mainly ATR/CATR-mediated) injury according to *in vitro* studies using rat and human hepatocytes (Yin et al. 2008). Regarding ATR/CATR toxicity, glycyrrhizic acid, a ROS neutralizer, presumably prevents lipid peroxidation and other negative consequences of oxidative stress. In turn, puerarin, an isoflavone glycoside that merges antioxidant and MPT blockade properties, may also provide hope to patients intoxicated by *X. strumarium* (Keskin Alkaç et al. 2022).

Strikingly, plant mitochondria seem to be less sensitive to CATR, as the inhibition of AAC by this glycoside is largely relieved (almost entirely) by a sufficient ADP content based on studies using potatoes (Vignais et al. 1976); a similar effect is not observed in other organisms, such as mammals, as neither ADP nor ATP oppose the effects of CATR (Vignais et al. 1971, 1973). Therefore, the treatment of ATR/CATR intoxication is still limited to supportive care for symptomatic treatment, e.g., gastric lavage and activated charcoal cure; thus, the identification of a potent antitoxin has remained a goal for years (Georgiou et al. 1988; Stewart and Steenkamp 2000; Daniele et al. 2005; Turgut et al. 2005; Ozturk et al. 2021; Kececi et al. 2022; Keskin Alkaç et al. 2022).

The susceptibility to ATR/CATR substantially varies within individual species and among different species and is independent of sex, whereas the route of administration plays a role (Stuart et al. 1981; Krejci and Koechel 1992; Koechel and Krejci 1993; Mendez et al. 1998; Obatomi and Bach 1998; Yin et al. 2008; Bouabid et al. 2019). Although ATR/CATR are assumed to be noncumulative toxicants, their contents in the liver increase over time if these glycosides are regularly consumed (Mendez et al. 1998; Jiang, Yang, Xing, Yan, Guo, Hou, et al. 2019). Specifically, the leaching rate of ATR/CATR from ingested plant parts in herbivores, the microbiota-dependent preabsorptive degradation of these phytochemicals in the digestive system, and the efficacy of the hepatic enzymes metabolizing/detoxifying *ent-*kaurane glycosides, such as cytochrome P-448-dependent enzymes, affect morbidity and possibly mortality (Hatch et al. 1982; Witte et al. 1990; Obatomi and Bach 1998; Botha et al. 2014). Moreover, the low pH of gastric juice may contribute to ATR/CATR decomposition (Chen et al. 2013). Scant information regarding ATR/CATR excretion is available, but the kidneys seem to play a key role (Koechel and Krejci 1993; Obatomi et al. 1998, 2001; Obatomi and Bach 1998).

Curiously, ATR and CATR display comparable properties in terms of their absorption rate and elimination half-life (studies using rats) (Pan et al. 2020). However, the contents of these glycosides and their proportions in crude versus processed herbal material may considerably differ and are related to the geographical location (effects of climate and soil composition), harvesting time (effects of aging and desiccation), and genetic factors (Santi 1958; Obatomi and Bach 1998; Daniele et al. 2005; Chen et al. 2013; Yang et al. 2013; Yu et al. 2013; Nikles et al. 2015; Su et al. 2016; Fan et al. 2019; Jiang, Yang, Xing, Yan, Guo, Yang, et al. 2019; Khan et al. 2020; Pan et al. 2020). Additionally, variation in sampling and detection methods explains the incredibly large discrepancies in the ATR/CATR concentrations among the different parts of a tested plant (described above for *Xanthium* spp.) (Cole et al. 1980; Witte et al. 1990; Scherer et al. 2009; Chen et al. 2013; Yang et al. 2013; Botha et al. 2014; Nikles et al. 2015; Machado et al. 2021; Ozturk et al. 2021; Kececi et al. 2022; Keskin Alkaç et al. 2022). In society, while it has been customarily assumed, researchers have scientifically proven that the correct processing of raw *Xanthium* burs containing fruits, especially when their extracts are orally consumed, alters the ATR/CATR contents to the benefit of the user (Kamboj and Saluja 2010; Chen et al. 2013; Yang et al. 2013; Yu et al. 2013; Nikles et al. 2015; Su et al. 2016; Jiang, Yang, Xing, Yan, Guo, Hou, et al. 2019; Jiang, Yang, Xing, Yan, Guo, Yang, et al. 2019). The drying of burs is often a prerequisite, but the removal of the external spines and achenes with seeds is not necessary. Stir frying or baking, followed by hydrothermal treatment, to prepare an infusion/decoction are the typical procedures for preparing a traditional remedy from complete burs. Notably, processed, e.g., roasted, *X. strumarium* burs, which should be initially ripe, intact, and dried, are generally less injurious because this approach may significantly decrease the content of the more poisonous CATR, whereas the ATR content may markedly increase (Nikles et al. 2015; Su et al. 2016; Jiang, Yang, Xing, Yan, Guo, Yang, et al. 2019). Under these conditions, CATR is reportedly decarboxylated to form ATR (Nikles et al. 2015). Nevertheless, random analyses of processed *Fructus xanthii* batches, e.g., baked according to the label, revealed the alarming finding that trade samples may still contain high concentrations of CATR and much lower levels of ATR (Nikles et al. 2015; Jiang, Yang, Xing, Yan, Guo, Yang, et al. 2019). Heating gently dried *X. strumarium* burs in an oven for 20–25 min at a temperature of 140–172°C decreases CATR to an undetectable level (Nikles et al. 2015). Autoclaving may also substantially deplete CATR (trace amount) in uncut burs of *X. strumarium* (Witte et al. 1990).

## Physiological functions of atractyloside and carboxyatractyloside

The heteroside CATR has at least three major native tasks. Presumably, this glycoside originally functioned as a plant growth regulator to delay seed germination (Cutler and Cole 1983). As an inhibitor responsible for dormancy, CATR contributes to survival under unfavorable environmental conditions. Typically, each bur of *Xanthium* spp. contains a pair of achenes, usually one smaller and one larger achene, although burs have been shown to bear 25 fruits, some of which are not fully developed (Wareing and Foda 1957; Löve and Dansereau 1959; Stuart et al. 1981; Cutler and Cole 1983; Weaver and Lechowicz 1983; Abbas et al. 1999; Turgut et al. 2005; Cesur et al. 2018; Khan et al. 2020; Keskin Alkaç et al. 2022; Ullah, Khan, Hewitt, et al. 2022). Smaller fruit, whose seed may take years to start sprouting, is often referred to as ‘upper' fruit when it is located slightly above the ‘lower' fruit in the locule of the bur (Cutler and Cole 1983; Weaver and Lechowicz 1983; Amini et al. 2020). In contrast, the seed of the larger and ‘inferior' achene typically germinates during the following spring and, thus, is nondormant. When the plant does not produce seeds during the current year because of biotic and/or abiotic stresses, the remaining seeds from the previous season allow the continuity of cocklebur populations to be preserved. The higher concentration of CATR in the ‘superior' seed may be responsible for its innate latency and, thus, the strategy of seed partitioning (Cutler and Cole 1983; Witte et al. 1990). Consequently, the elevated contents of ATR and CATR, which are water-soluble germination inhibitors, present in an embryo of the ‘upper' seed might drive the higher oxygen tension needed to finally overcome prolonged dormancy, e.g., through the oxidase-dependent breakdown of these blockers (Wareing and Foda 1957; Porter and Wareing 1974). The leaching of ATR and CATR, originally referred to as germination inhibitors ‘A' and ‘B', respectively, from hydrated seeds through a disorganized testa, the true seed coat, was proposed to facilitate seed sprouting (Wareing and Foda 1957; Porter and Wareing 1974; Cutler and Cole 1983; Witte et al. 1990). The intact testa is rather impermeable to endogenous developmental inhibitors and establishes a barrier that trammels the oxygen supply to the seeds (Wareing and Foda 1957; Porter and Wareing 1974). In nature, both the rupture of the seed coat and adequate oxygen pressure may contribute to overcoming the latency sustained by ATR/CATR.

Similarly, cyanide, such as hydrogen cyanide (HCN), an inhibitor of a terminal oxidase in the mitochondrial electron transport chain, known as complex IV, that affects mitochondrial respiration, may regulate, i.e., inhibit or stimulate, germinability in a concentration-dependent manner (Esashi et al. 1991; Siegień and Bogatek 2006). HCN is produced in certain plant species, including *Xanthium* spp., during processes, such as the catabolism of cyanogenic glycosides and cyanogenic lipids. Accordingly, in the rhizomes of *A. gummifera,* the ATR content is increased during the winter (Daniele et al. 2005), which likely helps maintain the plant in a resting state until spring. Therefore, compounds that are extremely toxic to animals and humans have crucial modulatory functions in the ontogenesis of many eukaryotic autotrophs. In addition to ATR/CATR and HCN, the expression level of the delay of germination 1 (*dog1*) gene, which protein product, among others, indirectly influences the cell wall properties, and some respiration-associated genes, which protein products are indirectly responsible for a potentially high level of energy (ATP) production and, thus, biosynthesis (Nemati et al. 2020, 2022), a burial depth of achenes or seeds, where 15–18 cm may constitute a critical suppression threshold with no seedling emergence, and the amount of mulch (Amini et al. 2020; Saeed et al. 2020) affect the prolonged dormancy or its lack in dimorphic seeds of *X. strumarium*.

The effects of ATR/CATR leached from cockleburs on the environment are not neutral. Analogous to the delayed development of the parent plants from *Xanthium* seeds, which tightly maintain ATR/CATR reserves, the released deposits of these glycosides might play another convergent role in the wild. The secondary effect is the provision of a habitat niche maintained by growth inhibitors, e.g., targeting competing plant species. This scenario might result not exclusively from washing ATR/CATR from seeds to disrupt dormancy (Cutler and Cole 1983). The infusion of soil with these defensive glycosides may constantly occur *via* an unknown mechanism or the leaching of ATR/CATR from plant residues from previous years during the normal decomposition process (Kadioglu 2004). Thus, ATR/CATR producers, such as *Xanthium* spp., could obtain a large advantage, such as access to the appropriate amount of light across vegetation periods, for maintaining a stand in an ecosystem. This strategy might be similar to the effects of bacteria and fungi secreting antibiotics into the soil (de Boer et al. 2005). Interestingly, CATR or extracts of *X. strumarium* may exert negative or positive allelopathic effects on some crops and weeds; thus, their potential use as natural biocides/herbicides is limited (Cutler and Cole 1983; Kadioglu 2004). Notably, CATR is unstable in protic solvents, such as methanol, but high concentrations of ATR and CATR can be retained in the roots of *A.* *gummifera* purchased from a Moroccan herbalist for as long as seven years after drying (Carlier et al. 2014).

The negative allelochemical nature of ATR/CATR may affect not only adjacent plants but also animals, particularly to discourage feeding by herbivores (Figure 3). The deployment of a mechanical ‘weapon', i.e., the mature spiny involucre usually encapsulating two achenes in *Xanthium* spp. (Figure 2), along with a chemical ‘weapon', such as various phytotoxins, certainly more efficiently deter phytophages. Unsurprisingly, in very young plants without burs, CATR is concentrated in cotyledonary leaves (Rostafiński and Seidl 1973; Cole et al. 1980; Stuart et al. 1981; Scherer et al. 2009; Gurley et al. 2010; Botha et al. 2014; Rice et al. 2019; Barbosa et al. 2020; Machado et al. 2021). Cocklebur bristles also facilitate an epizoochoric mechanism of dispersion, and hydrochory is promoted by the considerable buoyancy of burs, which can employ spikes to trap air bubbles (Löve and Dansereau 1959; Weaver and Lechowicz 1983; Kamboj and Saluja 2010; Galanos 2015; Mueller-Bieniek et al. 2015; Rozina et al. 2017; Machado et al. 2021; Müller-Kiefer and Tomasello 2022; Ullah, Khan, Hewitt, et al. 2022). Naked achenes are also buoyant as they possess aerated tissues (Mueller-Bieniek et al. 2015).

## Laboratory detection and use of atractyloside and carboxyatractyloside

In addition to causing accidental or inadvertent deaths, solutions containing ATR and CATR have been utilized to induce abortion and commit suicide or even homicide (Gaillard and Pepin 1999; Stewart and Steenkamp 2000; Daniele et al. 2005; Carlier et al. 2014; Bouabid et al. 2019). However, persons with such ill intentions cannot rest easily. Modern, simple, sensitive and rapid methods of screening and quantifying ATR/CATR levels in blood/serum, urine, gastric contents, and liver samples using, e.g., high-performance liquid chromatography coupled with high-resolution tandem mass spectrometry (HPLC–HRMS/MS), ultra-performance liquid chromatography with tandem mass spectrometry (UPLC–MS/MS), and gas chromatography–mass spectrometry (GC–MS) have been developed for potential application in clinical and forensic medicine (Carlier et al. 2014; Jiang, Yang, Xing, Yan, Guo, Hou, et al. 2019; Pan et al. 2020; Ozturk et al. 2021; Kececi et al. 2022; Keskin Alkaç et al. 2022; Roh et al. 2022). These precise and flexible methods were preceded by similar or other types of reliable detection techniques for ATR/CATR or their monodesulfated derivatives (Gaillard and Pepin 1999; Stewart and Steenkamp 2000; Steenkamp et al. 2004, 2006). Enzyme immunoassays with antibodies against ATR and the thin-layer chromatography (TLC) spot tests may also be helpful for short-term analyses of contaminated urine and other samples (Bye et al. 1990; Steenkamp et al. 1999; Stewart and Steenkamp 2000). Advanced measurement approaches may not only contribute to pathophysiological analyses but also indicate the storage sites of these glycosides in plants (Roeder et al. 1994; Steenkamp et al. 2004, 2006; Scherer et al. 2009; Yang et al. 2013; Botha et al. 2014; Nikles et al. 2015; Su et al. 2016; Jiang, Yang, Xing, Yan, Guo, Hou, et al. 2019; Jiang, Yang, Xing, Yan, Guo, Yang, et al. 2019).

Based on the presumed high specificity of CATR for AAC (Klingenberg 2008), this compound is among the most popular and interesting inhibitors used in mitochondrial research. Distinctly, CATR has been administered to block OXPHOS, i.e., AAC-mediated ADP/ATP turnover, which is considered the most common standard application, and limit AAC-perpetrated mitochondrial proton (H^+^) leak in studies using isolated mitochondria and/or mitoplasts (Figure 4(b)) (Andreyev et al. 1989; Echtay et al. 2003; Brand et al. 2005; Klingenberg 2008; Woyda-Ploszczyca and Jarmuszkiewicz 2014a; Bertholet et al. 2019). Proton conductance across the IMM from the intermembrane space (IS) to the mitochondrial matrix but beyond F_O_F_1_-ATP synthase is an example of an energy dissipation mechanism (Figure 4(b)) (Woyda-Ploszczyca and Jarmuszkiewicz 2017). Normally, the energy-conserving pathway mediated by F_O_F_1_-ATP synthase consumes most of the mitochondrial electrochemical H^+^ gradient, which is generated by respiratory chain pumps (Figure 4(a)). AAC is not the only protein that contributes to ‘futile' H^+^ uptake in mitochondria. A typical carrier protein in the IMM involved in the short circuiting of H^+^ chemiosmosis in this organelle is uncoupling protein (UCP) (Figure 4(b)). Therefore, both AAC- and UCP-mediated unidirectional H^+^ transfer result in OXPHOS uncoupling. This function of AAC/UCP decreases ATP synthesis during aerobiosis, i.e., ATP production coupled with oxygen-dependent respiration is disturbed. Nevertheless, mild (partial) protein-dependent uncoupling might be beneficial because it contributes to maintaining the redox balance of the electron transport chain, thus counteracting the risk of elevated free radical species egress. The phospholipid bilayer portion of the IMM and, therefore, its fatty acyl composition usually plays a marginal role in H^+^ translocation (Brand et al. 2005). The natural modulators considered stimulators of uncoupling *via* AAC/UCP are membrane long-chain free fatty acids (FFAs), such as linoleic and palmitic acid (Figure 4(a)) (Andreyev et al. 1989; Woyda-Ploszczyca and Jarmuszkiewicz 2014a, 2017), and some aldehydes, particularly HNE (Figure 4(b)) (Echtay et al. 2003; Woyda-Ploszczyca and Jarmuszkiewicz 2012, 2013, 2014b, 2017). However, the metabolite P-CoA blocks AAC dissipating activity (Andreyev et al. 1989), while purine ribonucleoside di- and triphosphates (PNs), such as GDP and GTP, inhibit UCP (Figures 1 and 4(a)) (Echtay et al. 2003; Parker et al. 2008; Woyda-Ploszczyca and Jarmuszkiewicz 2011, 2012, 2013, 2014a, 2014b, 2017). Regarding the effectiveness of GDP and GTP in hindering UCP, GTP is a relatively stronger, more specific, and more ‘diagnostic' (physiological) blocker than GDP in different organisms (Woyda-Ploszczyca and Jarmuszkiewicz 2011, 2014a, 2017). Unfortunately, no reports concerning the magnitude of mitochondrial H^+^ leak in CATR-synthesizing plants are available, which might naturally use this glycoside to negatively regulate uncoupling *via* AAC. Nevertheless, AAC-mediated H^+^ conductance might occur without obstacles in the mitochondria of plants, such as cockleburs, because even CATR-inhibited AAC may be partially implicated in H^+^ leak (Brand et al. 2005). Curiously, ATR, such as oligomycin (Figure 1), does not cause a collapse of the cell and mitochondrial membrane potentials (Andersson et al. 1987), and CATR favors an increase in the mitochondrial membrane potential in isolated mitochondria (Echtay et al. 2003; Brand et al. 2005; Parker et al. 2008; Woyda-Ploszczyca and Jarmuszkiewicz 2014a). Therefore, the inhibition of AAC-catalyzed H^+^ leak by ATR/CATR, which are exogenous and toxic substances for most organisms, may reflect the integrity of the mitochondria to some degree and allow us to evaluate the contribution of nonphosphorylating H^+^ uptake to apparent respiration and the subsequent metabolic rate.

The use of CATR at low concentrations may allow the quantification of functional AACs (Brandolin et al. 1980; Streicher-Scott et al. 1993; Brand et al. 2005). The determination of the content of this translocator, namely, its active pool, from the CATR titer is usually based on the stoichiometric assumption that one molecule of the glycoside binds each AAC dimer (Klingenberg et al. 1978). In turn, radiolabeled ATR/CATR ([^3^H] or [^35^S]) have been exploited to (i) confirm the presence of competent AAC in the research milieu, (ii) test the mechanisms regulating AAC kinetics (inhibitor-carrier protein, substrate-inhibitor, and inhibitor-other inhibitor interactions) (Klingenberg et al. 1971; Vignais et al. 1971, 1973, 1976; Riccio et al. 1973; Scherer et al. 1973; Brandolin et al. 1980), and (iii) apply a ligand that serves as an indicator/stabilizer of purified and undenatured AAC (Klingenberg et al. 1978). The isolation of AAC-ATR or AAC-CATR complexes followed by their crystallization enables the projection of the spatial structure of this carrier protein at a high resolution and the exploration of the specificity of substrate/toxin binding as ADP and ATR/CATR docking sites overlap (Kunji and Harding 2003; Pebay-Peyroula et al. 2003; Kedrov et al. 2010).

## Target proteins of atractyloside and carboxyatractyloside other than ADP/ATP carriers

**Figure 5.**
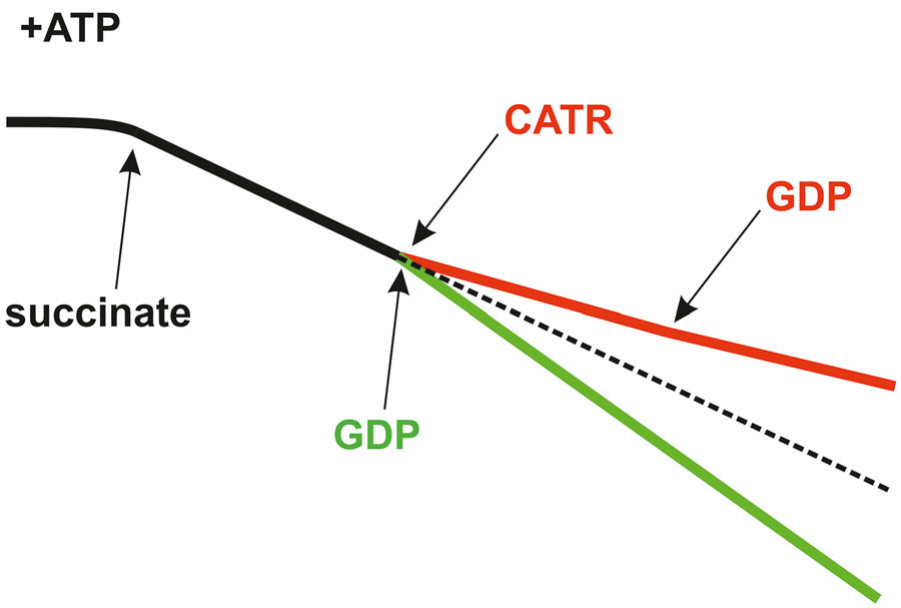
Inhibition of GDP metabolism in mitochondria by carboxyatractyloside (CATR). In mitochondria isolated from different sources, i.e., amoeba, yeast, potato and mammalian cells/tissues, including those from rat kidneys, CATR treatment inhibits GDP metabolism in the presence of ATP, mainly mtNDPK-sustained GDP transphosphorylation. Therefore, the schematic shows that basal mitochondrial respiration (black trace) is never increased (red trace) upon acute exposure to this glycoside followed by GDP addition. In contrast, in the absence of CATR, OXPHOS is induced after the application of GDP, which is observed as accelerated oxygen consumption (green trace) because mtNDPK generates an ADP pool, i.e., ATP + GDP → ADP + GTP. Solid/dashed black trace: conditions without CATR and exogenous ADP/GDP. In rat kidney mitochondria, 0.8 or 1 mM ATP and 1 mM GDP were used (Woyda-Ploszczyca and Jarmuszkiewicz 2014a). Succinate serves as an exogenous respiratory substrate in the absence of oligomycin. These types of traces can be recorded with Clark oxygen electrodes. O_2_ uptake values, which are usually reported in nanomoles O per minute per milligram of protein, are intentionally omitted as they may substantially differ depending on the species. The figure was created by the author with CorelDRAW.

Studies of isolated mammalian mitochondria, including rat kidney and human endothelial cell mitochondria, were the first to reveal that H^+^ leak, which is measured in the absence or presence of exogenous FFAs, is not curtailed but is stimulated by high concentrations of GDP (up to 1 mM) (Figure 5) (Woyda-Ploszczyca and Jarmuszkiewicz 2014a). The stimulatory effect of GDP was observed only in the absence of the OXPHOS inhibitors CATR and oligomycin (Figures 1 and 5) and in the presence of a sufficiently high concentration of exogenous ATP (0.8 or 1 mM). This feature might be evolutionarily well conserved as it occurs in isolated amoeba, yeast, and potato mitochondria (Woyda-Ploszczyca and Jarmuszkiewicz 2017, 2019). Considering the previous results obtained after a small amount of GDP was added to respiring and coupled mitochondria (Pedersen 1973; Jacobus and Evans 1977; Valenti et al. 1999), the involvement of mitochondrial nucleoside diphosphate kinase (mtNDPK; EC number 2.7.4.6) provides an explanation for the stimulatory effect of high concentrations of GDP. These novel findings of efficient mtNDPK activity in the presence of 1 mM GDP are intriguing (Woyda-Ploszczyca and Jarmuszkiewicz 2014a, 2017, 2019). When this enzyme was previously tested in isolated mitochondria from mammals, its partial inhibition was observed in the presence of much lower concentrations of GDP, i.e., greater than 0.15 mM, and 0.6 mM GDP completely blocked the activity of mtNDPK (Pedersen 1973; Valenti et al. 1999). NDPK catalyzes a transphosphorylation reaction, the exchange of a γ-phosphate group between donor nucleoside triphosphate and acceptor nucleoside diphosphate, e.g., ATP + GDP → ADP + GTP (Pedersen 1973; Tokarska-Schlattner et al. 2008). Although NDPK exhausts valuable ATP, it produces precious GTP. The ubiquitous presence of NDPK homologs, including cytosolic and mitochondrial isoforms, indicates their crucial roles in metabolism, cellular homeostasis and proper development (Takács-Vellai et al. 2015; Lacombe et al. 2018). For example, different steps in mitochondrial gene expression, including RNA and protein synthesis and mitochondrial Fe-S cluster (cofactor for proteins, such as aconitase, the key enzyme in the TCA cycle) biogenesis, require GTP, which may be delivered by mtNDPK residing in the mitochondrial matrix (Amutha et al. 2008). Additional sources of GTP in this organelle depend on the species and are determined by (i) the TCA cycle as succinyl-coenzyme A synthetase may form GTP, (ii) OXPHOS, which may produce GTP because F_O_F_1_-ATP synthase is able to phosphorylate GDP, or (iii) GTP direct import into mitochondria by specific carrier proteins (Vozza et al. 2004; Woyda-Ploszczyca and Jarmuszkiewicz 2014a). In mammals, the NDPK-D variant is the only isoform targeting mitochondria (Tokarska-Schlattner et al. 2008; Lacombe et al. 2009, 2021; Schlattner et al. 2013; Zala et al. 2017). This kinase is a peripheral membrane protein directed toward the IS and matrix and, thus, is bound to both sides of the IMM through electrostatic interactions, principally to cardiolipin, the dominant anionic phospholipid of this membrane (Figure 4). Interestingly, AAC and mtNDPK are presumably colocalized in cardiolipin patches in the IMM.

The rapid GDP metabolism observed in mitochondria *in vitro*, even at high GDP concentrations, is unsurprising considering the functional cooperation of mtNDPK and AAC (Woyda-Ploszczyca and Jarmuszkiewicz 2014a, 2017, 2019; Zala et al. 2017; Atlante and Valenti 2021). Under physiological-like conditions, i.e., without OXPHOS inhibitors but with saturated ATP and GDP concentrations, mtNDPK immediately transforms GDP into GTP at the expense of ATP, thus generating an ADP pool that is ultimately responsible for OXHPOS induction recorded as the stimulation of respiration during oxygraphic measurements (Figure 5). In fact, GDP is only an indirect inducer of OXPHOS; GDP regenerates free mtNDPK by accepting phosphate donated to the enzyme *via* ATP as a part of a ‘ping-pong' mechanism involving the phosphoenzyme intermediate (Biondi et al. 1998). Therefore, mtNDPK activity promotes OXPHOS and simultaneously considerably decreases the magnitude of protein-mediated mitochondrial H^+^ leak (Figure 4(a)). This occurs because AAC is primarily involved in ADP/ATP turnover during OXPHOS, and UCP action is markedly constricted as the GTP (considered the robust physiological inhibitor of UCP) concentration increases when mtNDPK is active. Moreover, intensive OXPHOS decreases the electrochemical H^+^ gradient; thus, the opportunity for AAC/UCP to uncouple is substantially limited. Consequently, the energy-consuming processes of the cell are efficiently powered.

How does CATR exposure change the local ADP/ATP and GDP/GTP concentrations, which are normally controlled by proteins, such as AAC and mtNDPK, and the overall functioning of animal and human mitochondria? The situation in the IS is described first as both CATR, primarily interacting with AAC, and PNs, such as GTP and GDP, inhibiting UCP, bind their targets from the cytosolic face of the IMM (Klingenberg 2008; Woyda-Ploszczyca and Jarmuszkiewicz 2011, 2017). Holistically, CATR affects PN turnover and channeling because when AAC is inhibited, a negative domino effect occurs, especially on proteins hydrolyzing or synthesizing ATP (Figure 4(b)). If AAC and mtNDPK are colocalized but not necessarily directly associated (Knorpp et al. 2003; Tokarska-Schlattner et al. 2008; Lacombe et al. 2009; Schlattner et al. 2013; Zala et al. 2017), which might also apply to AAC and UCP (Echtay et al. 2003; Parker et al. 2008; Woyda-Ploszczyca and Jarmuszkiewicz 2014a, 2017), CATR indirectly influences the neighboring catalysts of transphosphorylation, such as mtNDPK, and H^+^ leak, such as UCP. By extrapolation, the close localization of mtNDPK, AAC and UCP results in a regulatory interplay between the kinase and the protein-mediated ‘futile' H^+^ conductance (Figure 4(a)). The inhibition of AAC by CATR synchronously excludes the principal function, i.e., ADP/ATP antiport, and, to some extent, the additional function of this translocase, such as participation in H^+^ leak (Table 1) (Andreyev et al. 1989; Echtay et al. 2003; Brand et al. 2005; Klingenberg 2008; Woyda-Ploszczyca and Jarmuszkiewicz 2014a; Bertholet et al. 2019). The observed sensitivity of UCP to CATR may result from the trapping of AAC-UCP heterodimers (activated exogenously or not) capable of conducting H^+^; thus, presumably, the indirect inhibition of UCP by this glycoside remains to be determined (Figure 4(b) and Table 1) (Echtay et al. 2003; Parker et al. 2008; Woyda-Ploszczyca and Jarmuszkiewicz 2014a, 2017). Consequently, although some substrates for mtNDPK are present in the IS at high concentrations, such as GDP, the CATR-induced severe shortage of critical ATP indirectly inactivates this kinase, and ADP molecules from various sources, including those generated by mtNDPK, accumulate in the IS (Figure 4). Indeed, inactive mtNDPK in the IS neither drives OXPHOS (Figures 4 and 5) nor counteracts energy dissipation *via* UCP; thus, the disruption of GTP (considered the physiologically relevant inhibitor of UCP) production *via* mtNDPK in the IS results in further energy losses (Figure 4). Not solely ATP is depleted as an effect of the indirect blockade of F_O_F_1_-ATP synthase by CATR because the quick secondary response to this glycoside and plant extracts containing CATR is the promotion of oxidative stress (Wang et al. 2011; Keskin Alkaç et al. 2022). Curiously, exogenous treatment with the reactive aldehyde HNE, the possible lipid peroxidation end product, stimulates H^+^ leak *via* AAC/UCP, e.g., in mitochondria isolated from rat kidneys and livers (Echtay et al. 2003; Woyda-Ploszczyca and Jarmuszkiewicz 2012, 2014b, 2017). The lack of ADP entry into the matrix maintains a high H^+^ gradient across the IMM, which favors uncoupling, as the activity of F_O_F_1_-ATP synthase, a desirable consumer of the H^+^ gradient for energy conservation, is substantially limited in the presence of OXPHOS inhibitors, including CATR. Under these conditions, the H^+^ leak mediated by UCP might also be enhanced by the depletion of GTP in the IS, which is usually mainly delivered by mtNDPK as its final product because potentially more available inhibitory GDP often shows less specificity for this carrier protein (Woyda-Ploszczyca and Jarmuszkiewicz 2011, 2014a, 2017). However, this HNE-induced AAC/UCP-dependent mild uncoupling mediated by a negative feedback loop (Figure 4(b)) (Echtay et al. 2003; Woyda-Ploszczyca and Jarmuszkiewicz 2017) probably evolved to manage unfavorable situations, such as contact with toxins affecting OXPHOS, lasting only for a relatively short period and forcing the uptake of a low concentration of the xenobiotic. The transient discomfort of the organism indicates slight poisoning, with a minor proportion of the AAC pool saturated with CATR. Therefore, the unloading of the H^+^ gradient with the help of uninhibited AAC/UCP might provide the time needed to regenerate active AAC for ADP/ATP antiport *via* compensatory expression. Hydroxynonenal-stimulated uncoupling in mitochondria may occur under phosphorylating conditions (Woyda-Ploszczyca and Jarmuszkiewicz 2013), supporting the hypothesis that organelles fight to survive in response to a CATR-induced increase in the HNE level. The efficacy of this type of first-line antioxidant defense, i.e., prevention of respiratory chain overreduction and downstream detrimental effects, such as excessive ROS generation/lipid peroxidation, depends on the relative concentrations of effectors alleviating, e.g., CATR, and factors driving, e.g., HNE, H^+^ translocation through AAC and UCP across IMM. Unfortunately, in the case of severe poisoning, this process is rather ineffective and only prolongs the onset of imminent destruction because the underlying problem still exists in the mitochondrial matrix, where the catalytic portion of F_O_F_1_-ATP synthase and the TCA cycle enzymes are seriously disabled when ATR/CATR indirectly but substantially limit oxygen uptake. Finally, the protracted lack of ATP delivery from mitochondria contributes to cell death and organ damage, including early CATR/*Xanthium* spp. intoxication symptoms, such as hepatorenal syndrome in animals and humans (Stewart and Steenkamp 2000; Daniele et al. 2005; Turgut et al. 2005; Machado et al. 2021; Keskin Alkaç et al. 2022). In the proposed model, the extent of tissue damage and, thus, the general outcome of poisoning certainly depends on the ATR/CATR dosage. Importantly, this comprehensive scenario is partly but clearly supported by the results obtained in a study of isolated rat kidney mitochondria treated with and without exogenous activators of H^+^ leak, i.e., FFAs, under conditions promoting/preventing OXPHOS pathway and mtNDPK-catalyzed reaction (Woyda-Ploszczyca and Jarmuszkiewicz 2014a). The indirect inhibition of mtNDPK also occurred in the presence of oligomycin (0.7 μg/mL). Conversely, the direct inhibition of mtNDPK by CATR (3.6 μM) should also be considered. Mitochondrial nucleoside monophosphate kinase (NMPK) and mtNDPK were shown to be sensitive to ATR through the potential binding of this glycoside (Table 1) (Allmann et al. 1967). Thus, ATR/CATR not only limit access to ATP but may also block mtNDPK activity in a more specific manner; however, this issue has been ignored in numerous CATR poisoning analyses. Nevertheless, functional studies using isolated mitochondria are insufficient to establish the latter possibility because of the potential overlap of AAC and mtNDPK inhibition by CATR (Figure 5) (Woyda-Ploszczyca and Jarmuszkiewicz 2014a, 2017, 2019). Another piece of evidence explaining the strong toxicity of CATR in the kidney and liver is the remarkably high expression of mtNDPK in these organs, which is higher in the kidney than in other tissues (Lacombe et al. 2009).

As mentioned above, the TCA cycle is indirectly but negatively affected by ATR/CATR (Table 1) (Santi 1958; Xue et al. 2014). Interestingly, the IMM tricarboxylate (citrate) carrier is directly inhibited by ATR/CATR, which has also been completely overlooked (Table 1) (Shug and Shrago 1973; Morel et al. 1974). In turn, the relevant indirect targets of these glycosides, which are likewise shrouded in oblivion, include mitochondrial F_O_F_1_-ATP synthases/ATPases, such as those from the kidneys (Table 1) (Kinne-Saffran and Kinne 1979; Ebel and Ruf 1985; Woyda-Ploszczyca and Jarmuszkiewicz 2014a). Therefore, multiple, often initially ‘concealed' for us targeting sites, both direct and indirect, are involved in the harmful effect of ATR/CATR on mitochondria *in vivo* (Table 1). Essentially, the real cascade of ADP/ATP-requiring enzyme inhibition is mediated to some extent by the AAC pool deterioration resulting from ATR/CATR docking at these antiporters.

## Conclusions

Accordingly, the use of indigenous/imported medicines possibly containing ATR/CATR and the application of these glycosides, e.g., during mitochondrial H^+^ leak studies, constitute a real dilemma. *Xanthium*-based remedies from processed plant materials are important for TCM and have been commonly introduced into use, mainly in Asian countries, including in clinical practice (Yu et al. 2013; Fan et al. 2019; Sheng et al. 2019; Khan et al. 2020; Chen et al. 2021). Nevertheless, techniques for eliminating ATR/CATR must be refined to meet safety requirements and exclude biohazards. A constant risk of accidental poisoning exists because wild cockleburs can grow directly on the beach even in popular European resorts in the Mediterranean Sea, such as Faliraki on the Greek Island of Rodos, e.g., immediately next to water sports facilities, and have not been removed (Figure 2(c,d)). Therefore, in the past and present decade of the twenty first century, public awareness of the severe toxicity and invasiveness of cocklebur has been limited, and these characteristics have often been perceived by only narrow specialists in Europe (personal observations from Greece and Poland) and other continents (García et al. 2017; Iqbal et al. 2020, 2021; Jun et al. 2021; Machado et al. 2021; Roh et al. 2022; Ullah, Khan, Hewitt, et al. 2022). Farmers, silage and other types of feed producers, veterinarians, and persons working for environmental protection and monitoring agencies are often not familiar with the danger of *Xanthium* spp.; thus, informative programs and management strategies concerning cocklebur-related risks are urgently needed to limit deaths and economic losses. Similar approaches are needed for *Iphiona aucheri* (Boiss.) Anderb. (Asteraceae), which is listed in the FDA (2022) because of ATR/CATR synthesis, and which may unpredictably disrupt sporting events in some regions, such as the United Arab Emirates, when racing camels (*Camelus dromedarius*) in untreated pastureland are exposed to this plant and have accidental adsorption of toxic glycosides, followed by death (Roeder et al. 1994).

*Xanthium orientale* and *X. strumarium*, if taxonomically distinguished, are the most widely distributed invasive plants in not only Europe but also worldwide, except for Antarctica, and endanger the diversity and composition of native flora and many crops where cockleburs cause yield losses, e.g., in maize cultivation (Wolski et al. 2016; Seifu et al. 2017; Grădilă and Jalobă 2018; Abdessemed et al. 2020; Amini et al. 2020; Iqbal et al. 2020, 2021; Kelečević et al. 2020; Pereira Coutinho et al. 2021; Shkondrov et al. 2021; Tokarska-Guzik et al. 2021; Ullah, Khan, Ali, et al. 2021; Ullah, Khan, and Rahman 2021; Zhang et al. 2021; Müller-Kiefer and Tomasello 2022; Ullah, Khan, Ali, 2022; Ullah, Khan, Hewitt, et al. 2022). Interestingly, the presence of *X. orientale* may contribute to the decline of *X. strumarium* in a natural environment (Müller-Kiefer and Tomasello 2022), and mycoherbicides treatment and natural enemies release, such as polyphagous insect herbivores, that do not affect crop plants may help limit *Xanthium* spp. populations (Abdessemed et al. 2020; Iqbal et al. 2021). Simultaneously, the abundant bioproducts in *X. strumarium* might be a source of inexpensive medicinal substances targeting bacteria, fungi, and cancer cells, and harvesting cockleburs would decrease its global population and reconcile/integrate the interests of different social groups (Al-Mekhlafi et al. 2017; Ghahari et al. 2017; Kozuharova et al. 2019; Sultana et al. 2019; Khan et al. 2020; Tong et al. 2020; Shkondrov et al. 2021; Ullah, Khan, Hewitt, et al. 2022). Considering the antiviral properties of cockleburs, including possible support for HIV-1 therapy (Chen et al. 2021), it is tempting to speculate that in the near future, another pandemic, i.e., the global epidemic of SARS-CoV-2, could be eradicated with support from vital *X. strumarium* biomolecules. Moreover, the potential use of *X. strumarium* seed extract against mosquitoes has been recently reported (Al-Mekhlafi et al. 2017), and these insects are vectors of specific disease agents such as pathogen causing malaria (WHO 2022). Another benefit of *X. strumarium*, having the status of a synanthropic plant, is its phytoremediation potential for soil contaminated with diesel fuel or heavy metals (Sultana et al. 2019; Dib and Sadoudi Ali Ahmed 2020; Ullah, Khan, Ali, et al. 2021).

The available literature focusing on poisonings overlooks the fact that ATR/CATR not only negatively and directly influence AAC but also indirectly affect many other proteins, including UCP, in the mitochondria, and AAC and UCP are two major H^+^ leak catalysts (Figure 4(b) and Table 1) (Echtay et al. 2003; Parker et al. 2008; Woyda-Ploszczyca and Jarmuszkiewicz 2014a, 2017; Bertholet et al. 2019). Indirectly and/or directly, ATR/CATR also obviously negatively affect kinases implicated in the homeostasis of nucleotide metabolism, such as mtNDPK, and downstream processes, including the regulation of mitochondrial H^+^ gradient conversion and nucleic acid turnover (Table 1) (Woyda-Ploszczyca and Jarmuszkiewicz 2014a, 2017, 2019). Therefore, the use of ATR/CATR in studies of mitochondrial H^+^ leak/respiration aiming to describe the general mitochondrial physiology should be avoided, although these phytotoxins have played an invaluable role in the identification of energy transduction mechanisms in mitochondria. Observations from *in vitro* studies similar to undisturbed mitochondrial physiological conditions *in vivo* require OXPHOS-favoring conditions without inhibitors, such as ATR/CATR and oligomycin, and saturating concentrations of PNs to reliably register the interactions among orchestrated components.

Finally, the following three important main highlights arise from this work: (i) livestock may instinctively avoid eating *X. strumarium* in the field, (ii) CATR definitely inhibits ADP and GDP metabolism, and (iii) the direct/indirect targets of CATR are ambiguous, and other currently unknown, thus ‘silent' protein ‘victims' of this glycoside await identification.
